# (*Z*)-Ethyl 2-(4-chloro­phen­yl)-3-[(2,4-difluoro­phen­yl)amino]­prop-2-enoate

**DOI:** 10.1107/S1600536810043801

**Published:** 2010-10-31

**Authors:** Zhu-Ping Xiao, Xu-Dong Wang, Tian Liu, Jian Zhu, Zhi-Ping Li

**Affiliations:** a College of Chemistry and Chemical Engineering, Jishou University, Jishou 416000, People’s Republic of China

## Abstract

In the title compound, C_17_H_14_ClF_2_NO_2_, the amino­acrylo­yloxy group makes dihedral angles of 47.55 (11)° with the 4-chloro­phenyl group and 8.74 (12)° with the difluoro­phenyl group; the dihedral angle between the rings is 52.32 (11)°. The structure of the title compound reveals a *Z* configuration with respect to the C=C double bond in the amino­acrylate fragment. A bifurcated intramolecular N—H⋯(O,F) hydrogen bond occurs.  In the crystal, molecules are linked into chains by C—H⋯O hydrogen bonds.

## Related literature

For background to Schiff bases, see: You & Zhu, 2006 ▶. For applications of enamines, see: Xiao *et al.* (2007 ▶, 2008*a* ▶,*b* ▶,*c* ▶).
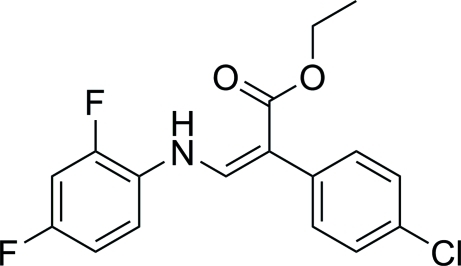


## Experimental

### 

#### Crystal data

C_17_H_14_ClF_2_NO_2_
*M* *_r_* = 337.74
Monoclinic, 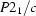

*a* = 16.276 (3) Å
*b* = 7.5030 (15) Å
*c* = 13.812 (3) Å
β = 111.11 (3)°
*V* = 1573.5 (5) Å^3^
*Z* = 4
Mo *K*α radiation
μ = 0.27 mm^−1^
*T* = 298 K
0.30 × 0.10 × 0.10 mm

#### Data collection

Bruker SMART CCD area-detector diffractometer
Absorption correction: ψ scan (North *et al.*, 1968 ▶) *T*_min_ = 0.923, *T*_max_ = 0.973
2957 measured reflections
2824 independent reflections
1566 reflections with *I* > 2σ(*I*)
*R*_int_ = 0.027

#### Refinement

*R*[*F* ^2^ > 2σ(*F* ^2^)] = 0.065
*wR*(*F* ^2^) = 0.170
*S* = 0.99
2824 reflections
213 parameters
H atoms treated by a mixture of independent and constrained refinement
Δρ_max_ = 0.26 e Å^−3^
Δρ_min_ = −0.29 e Å^−3^

### 

Data collection: *SMART* (Bruker, 2007 ▶); cell refinement: *SMART*; data reduction: *SAINT* (Bruker, 2007 ▶); program(s) used to solve structure: *SHELXS97* (Sheldrick, 2008 ▶); program(s) used to refine structure: *SHELXL97* (Sheldrick, 2008 ▶); molecular graphics: *SHELXTL* (Sheldrick, 2008 ▶); software used to prepare material for publication: *SHELXL97*.

## Supplementary Material

Crystal structure: contains datablocks global, I. DOI: 10.1107/S1600536810043801/bq2244sup1.cif

Structure factors: contains datablocks I. DOI: 10.1107/S1600536810043801/bq2244Isup2.hkl

Additional supplementary materials:  crystallographic information; 3D view; checkCIF report

## Figures and Tables

**Table 1:** Hydrogen-bond geometry (Å, °)

*D*—H⋯*A*	*D*—H	H⋯*A*	*D*⋯*A*	*D*—H⋯*A*
N1—H18⋯F1	0.83 (3)	2.29 (3)	2.674 (3)	108 (3)
N1—H18⋯O1	0.83 (3)	2.07 (3)	2.675 (4)	129 (3)
C6—H6⋯O1^i^	0.93	2.51	3.321 (4)	146

Symmetry code: (i) 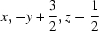.

**Fig. 1.**
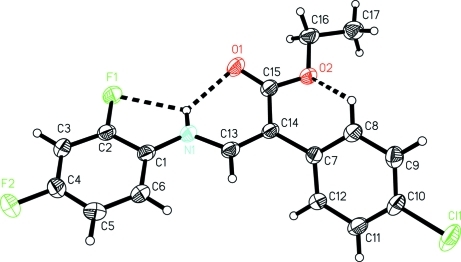
Molecular structure of the title compound, showing the atom-numbering scheme. Displacement ellipsoids are drawn at the 30% probability level.

**Fig. 2.**
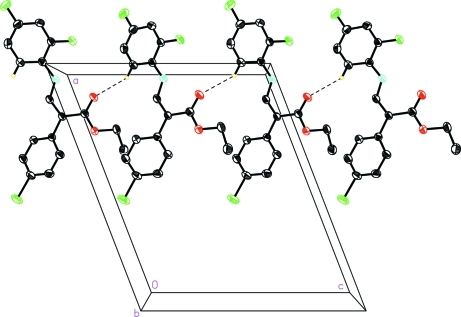
An infinite two-dimensional ribbon is formed through intermolecular C—H···O hydrogen bonds.

## supplementary crystallographic information

### Comment

A 2-aryl-3-arylaminoacrylate contains characteristic N—C═C bond and is therefore identified as enamine. It is well known that Schiff base harbors an N═C—C bond, which indicates that an enamine is the tautomeric isomer of the correspond Schiff base. Enamines, like Schiff bases (You & Zhu, 2006), show good antimicrobial activities (Xiao *et al.*, 2007; Xiao *et al.*, 2008*a*), especially against bacterium. On the other hand, an enamine is the key intermediate for anticancer agents, 3-arylquinolone (Xiao *et al.*, 2008*b*) and 3-arylquinoline (Xiao *et al.*, 2008*c*). In a continuation of our work on the structural characterization of enamine derivatives, we report herein the crystal structure of the title compound, (I).

The bond length of C13—N1 (1.344 (4) Å) is shorter than standard C—N single bond (1.48 Å) but longer than C—N double bond (1.28 Å), indicating that the *p* orbital of N1 is conjugated with the π molecular orbital of C13—C14 double bond. For the same reason, C1—N1 (1.394 (4) Å) is single bond with some double-bond character. The stereochemistry of the double bond in aminoacrylate moiety was assigned as (*E*)-configuration based on X-ray crystallography (Fig. 1) of the title compound.

Aminoacryloyloxy moiety, O2—C15—O1—C14—C13, forms a plane with the mean deviation of 0.0249 Å, which makes a dihedral angle of 47.55 (11) ° with the 4-chlorophenyl group and 8.74 (12) ° with the difluorophenyl group. The molecules are linked through intermolecular C—H···O hydrogen bonds, forming an infinite one-dimensional ribbons (Table 1, Fig. 2).

### Experimental

Equimolar quantities (6 mmol) of ethyl 2-(4-chlorophenyl)-3-oxopropanoate (1.36 g) and 2,4-difluorobenzenamine (0.77 g) in absolute alcohol (18 ml) were heated at 344–354 K for 2 h. The excess solvent was removed under reduced pressure. The residue was purified by a flash chromatography with EtOAc–petrolum ether (1:6, *v*/*v*) to afford two fractions. The second fraction gave a *E*-isomer, and the first fraction, after partial solvent evaporated, furnished colorless blocks of (I) suitable for single-crystal structure determination.

### Refinement

The H atom bonded to N1 was located in a difference Fourier map. All other H atoms were placed in geometrically idealized positions and constrained to ride on their parent atoms with C—H = 0.93, 0.96 and 0.97 Å for the aromatic, CH_3_ and CH_2_ type H atoms, respectively. *U*_iso_ = 1.2*U*_eq_(parent atoms) were assigned for aromatic and CH_2_ type H-atoms and 1.5*U*_eq_(parent atoms) for CH_3_ type H-atoms.

### Crystal data

**Table:**

C_17_H_14_ClF_2_NO_2_	*F*(000) = 696
*M**_r_* = 337.74	*D*_x_ = 1.426 Mg m^−^^3^
Monoclinic, *P*2_1_/*c*	Mo *K*α radiation, λ = 0.71073 Å
Hall symbol: -P 2ybc	Cell parameters from 1318 reflections
*a* = 16.276 (3) Å	θ = 1.8–24.7°
*b* = 7.5030 (15) Å	µ = 0.27 mm^−^^1^
*c* = 13.812 (3) Å	*T* = 298 K
β = 111.11 (3)°	Block, colourless
*V* = 1573.5 (5) Å^3^	0.30 × 0.10 × 0.10 mm
*Z* = 4	

### Data collection

**Table:**

Bruker SMART CCD area-detector diffractometer	2824 independent reflections
Radiation source: fine-focus sealed tube	1566 reflections with *I* > 2σ(*I*)
graphite	*R*_int_ = 0.027
φ and ω scans	θ_max_ = 25.3°, θ_min_ = 1.3°
Absorption correction: ψ scan (North *et al.*, 1968)	*h* = −19→18
*T*_min_ = 0.923, *T*_max_ = 0.973	*k* = −9→0
2957 measured reflections	*l* = 0→16

### Refinement

**Table:**

Refinement on *F*^2^	Primary atom site location: structure-invariant direct methods
Least-squares matrix: full	Secondary atom site location: difference Fourier map
*R*[*F*^2^ > 2σ(*F*^2^)] = 0.065	Hydrogen site location: inferred from neighbouring sites
*wR*(*F*^2^) = 0.170	H atoms treated by a mixture of independent and constrained refinement
*S* = 0.99	*w* = 1/[σ^2^(*F*_o_^2^) + (0.0804*P*)^2^] where *P* = (*F*_o_^2^ + 2*F*_c_^2^)/3
2824 reflections	(Δ/σ)_max_ < 0.001
213 parameters	Δρ_max_ = 0.26 e Å^−^^3^
0 restraints	Δρ_min_ = −0.29 e Å^−^^3^

### Special details

**Table:**

Geometry. All e.s.d.'s (except the e.s.d. in the dihedral angle between two l.s. planes) are estimated using the full covariance matrix. The cell e.s.d.'s are taken into account individually in the estimation of e.s.d.'s in distances, angles and torsion angles; correlations between e.s.d.'s in cell parameters are only used when they are defined by crystal symmetry. An approximate (isotropic) treatment of cell e.s.d.'s is used for estimating e.s.d.'s involving l.s. planes.
Refinement. Refinement of *F*^2^ against ALL reflections. The weighted *R*-factor *wR* and goodness of fit *S* are based on *F*^2^, conventional *R*-factors *R* are based on *F*, with *F* set to zero for negative *F*^2^. The threshold expression of *F*^2^ > σ(*F*^2^) is used only for calculating *R*-factors(gt) *etc*. and is not relevant to the choice of reflections for refinement. *R*-factors based on *F*^2^ are statistically about twice as large as those based on *F*, and *R*-factors based on ALL data will be even larger.

### Fractional atomic coordinates and isotropic or equivalent isotropic displacement parameters (Å^2^)

**Table:**

	*x*	*y*	*z*	*U*_iso_*/*U*_eq_	
C1	1.0185 (2)	0.7793 (5)	0.9700 (2)	0.0447 (9)	
C2	1.1023 (2)	0.7723 (5)	1.0451 (2)	0.0473 (9)	
C3	1.1764 (2)	0.8338 (5)	1.0316 (3)	0.0575 (10)	
H3	1.2316	0.8267	1.0841	0.069*	
C4	1.1653 (2)	0.9062 (6)	0.9370 (3)	0.0591 (10)	
C5	1.0852 (3)	0.9180 (5)	0.8598 (3)	0.0614 (11)	
H5	1.0799	0.9696	0.7966	0.074*	
C6	1.0122 (2)	0.8536 (5)	0.8756 (3)	0.0561 (10)	
H6	0.9575	0.8598	0.8222	0.067*	
C7	0.7054 (2)	0.6470 (5)	0.8669 (2)	0.0418 (8)	
C8	0.6325 (2)	0.7235 (5)	0.8805 (3)	0.0534 (10)	
H8	0.6377	0.7669	0.9456	0.064*	
C9	0.5528 (2)	0.7365 (5)	0.7997 (3)	0.0568 (10)	
H9	0.5046	0.7872	0.8103	0.068*	
C10	0.5451 (2)	0.6741 (5)	0.7036 (3)	0.0538 (10)	
C11	0.6148 (2)	0.5949 (5)	0.6881 (3)	0.0567 (10)	
H11	0.6087	0.5507	0.6229	0.068*	
C12	0.6946 (2)	0.5807 (5)	0.7697 (2)	0.0474 (9)	
H12	0.7418	0.5255	0.7590	0.057*	
C13	0.8638 (2)	0.7024 (5)	0.9292 (2)	0.0463 (9)	
H13	0.8524	0.7370	0.8610	0.056*	
C14	0.7936 (2)	0.6482 (5)	0.9515 (2)	0.0423 (8)	
C15	0.8066 (2)	0.5946 (5)	1.0578 (2)	0.0480 (9)	
C16	0.7412 (2)	0.4743 (6)	1.1713 (2)	0.0622 (11)	
H16A	0.7579	0.5751	1.2184	0.075*	
H16B	0.7856	0.3821	1.1972	0.075*	
C17	0.6536 (2)	0.4057 (6)	1.1638 (3)	0.0708 (12)	
H17A	0.6094	0.4936	1.1312	0.106*	
H17B	0.6540	0.3807	1.2321	0.106*	
H17C	0.6408	0.2985	1.1231	0.106*	
Cl1	0.44479 (7)	0.69755 (18)	0.60035 (8)	0.0887 (5)	
F1	1.10991 (12)	0.7013 (3)	1.13887 (14)	0.0654 (7)	
F2	1.23767 (15)	0.9700 (4)	0.92091 (18)	0.0856 (8)	
N1	0.94818 (18)	0.7123 (4)	0.9939 (2)	0.0483 (8)	
O1	0.87698 (16)	0.6036 (4)	1.13063 (17)	0.0660 (8)	
O2	0.73408 (15)	0.5283 (3)	1.06790 (16)	0.0521 (7)	
H18	0.961 (2)	0.673 (4)	1.054 (3)	0.050 (11)*	

### Atomic displacement parameters (Å^2^)

**Table:**

	*U*^11^	*U*^22^	*U*^33^	*U*^12^	*U*^13^	*U*^23^
C1	0.0458 (19)	0.047 (2)	0.0377 (18)	0.0000 (17)	0.0109 (15)	−0.0070 (17)
C2	0.047 (2)	0.056 (2)	0.0355 (18)	0.0045 (18)	0.0104 (15)	−0.0033 (17)
C3	0.041 (2)	0.077 (3)	0.049 (2)	−0.006 (2)	0.0093 (16)	−0.010 (2)
C4	0.052 (2)	0.070 (3)	0.062 (2)	−0.007 (2)	0.028 (2)	−0.011 (2)
C5	0.070 (3)	0.073 (3)	0.044 (2)	−0.002 (2)	0.024 (2)	0.001 (2)
C6	0.049 (2)	0.073 (3)	0.0389 (19)	−0.004 (2)	0.0072 (16)	−0.0010 (19)
C7	0.0405 (18)	0.043 (2)	0.0397 (18)	−0.0037 (16)	0.0115 (14)	0.0044 (16)
C8	0.047 (2)	0.063 (2)	0.045 (2)	0.0019 (19)	0.0100 (16)	−0.0049 (19)
C9	0.043 (2)	0.062 (3)	0.061 (2)	0.0066 (19)	0.0144 (18)	0.005 (2)
C10	0.045 (2)	0.055 (2)	0.046 (2)	−0.0034 (19)	−0.0015 (16)	0.0091 (18)
C11	0.058 (2)	0.065 (3)	0.0392 (19)	−0.005 (2)	0.0082 (17)	−0.0021 (19)
C12	0.0463 (19)	0.054 (2)	0.0399 (18)	0.0003 (18)	0.0130 (15)	−0.0008 (17)
C13	0.047 (2)	0.053 (2)	0.0334 (17)	0.0049 (18)	0.0082 (15)	0.0008 (16)
C14	0.0426 (19)	0.048 (2)	0.0317 (17)	0.0011 (17)	0.0079 (14)	−0.0009 (15)
C15	0.047 (2)	0.052 (2)	0.0404 (19)	−0.0026 (18)	0.0103 (16)	−0.0019 (17)
C16	0.069 (3)	0.077 (3)	0.0365 (19)	−0.007 (2)	0.0138 (18)	0.001 (2)
C17	0.075 (3)	0.081 (3)	0.058 (2)	−0.013 (3)	0.026 (2)	0.000 (2)
Cl1	0.0566 (7)	0.1127 (10)	0.0672 (7)	0.0014 (7)	−0.0135 (5)	0.0174 (7)
F1	0.0531 (12)	0.0978 (18)	0.0363 (11)	−0.0016 (12)	0.0051 (9)	0.0110 (12)
F2	0.0689 (15)	0.119 (2)	0.0838 (17)	−0.0253 (15)	0.0458 (13)	−0.0121 (16)
N1	0.0402 (17)	0.063 (2)	0.0353 (16)	−0.0006 (15)	0.0063 (13)	0.0050 (16)
O1	0.0500 (15)	0.099 (2)	0.0373 (14)	−0.0152 (15)	0.0018 (12)	0.0058 (14)
O2	0.0481 (14)	0.0676 (17)	0.0374 (12)	−0.0045 (13)	0.0116 (10)	0.0026 (12)

### Geometric parameters (Å, °)

**Table:**

C1—C2	1.385 (4)	C10—C11	1.364 (5)
C1—C6	1.387 (5)	C10—Cl1	1.747 (3)
C1—N1	1.394 (4)	C11—C12	1.384 (5)
C2—F1	1.365 (4)	C11—H11	0.9300
C2—C3	1.365 (5)	C12—H12	0.9300
C3—C4	1.366 (5)	C13—N1	1.344 (4)
C3—H3	0.9300	C13—C14	1.348 (5)
C4—C5	1.357 (5)	C13—H13	0.9300
C4—F2	1.362 (4)	C14—C15	1.463 (4)
C5—C6	1.371 (5)	C15—O1	1.225 (4)
C5—H5	0.9300	C15—O2	1.333 (4)
C6—H6	0.9300	C16—O2	1.449 (4)
C7—C12	1.383 (4)	C16—C17	1.484 (5)
C7—C8	1.390 (5)	C16—H16A	0.9700
C7—C14	1.489 (4)	C16—H16B	0.9700
C8—C9	1.377 (4)	C17—H17A	0.9600
C8—H8	0.9300	C17—H17B	0.9600
C9—C10	1.370 (5)	C17—H17C	0.9600
C9—H9	0.9300	N1—H18	0.83 (3)
			
C2—C1—C6	116.0 (3)	C10—C11—H11	120.2
C2—C1—N1	118.7 (3)	C12—C11—H11	120.2
C6—C1—N1	125.3 (3)	C7—C12—C11	121.1 (3)
F1—C2—C3	118.6 (3)	C7—C12—H12	119.4
F1—C2—C1	117.0 (3)	C11—C12—H12	119.4
C3—C2—C1	124.4 (3)	N1—C13—C14	127.8 (3)
C2—C3—C4	116.4 (3)	N1—C13—H13	116.1
C2—C3—H3	121.8	C14—C13—H13	116.1
C4—C3—H3	121.8	C13—C14—C15	119.0 (3)
C5—C4—F2	119.4 (4)	C13—C14—C7	118.7 (3)
C5—C4—C3	122.5 (4)	C15—C14—C7	122.4 (3)
F2—C4—C3	118.1 (4)	O1—C15—O2	122.4 (3)
C4—C5—C6	119.6 (4)	O1—C15—C14	124.3 (3)
C4—C5—H5	120.2	O2—C15—C14	113.2 (3)
C6—C5—H5	120.2	O2—C16—C17	107.1 (3)
C5—C6—C1	121.1 (3)	O2—C16—H16A	110.3
C5—C6—H6	119.5	C17—C16—H16A	110.3
C1—C6—H6	119.5	O2—C16—H16B	110.3
C12—C7—C8	117.7 (3)	C17—C16—H16B	110.3
C12—C7—C14	120.8 (3)	H16A—C16—H16B	108.6
C8—C7—C14	121.3 (3)	C16—C17—H17A	109.5
C9—C8—C7	121.3 (3)	C16—C17—H17B	109.5
C9—C8—H8	119.3	H17A—C17—H17B	109.5
C7—C8—H8	119.3	C16—C17—H17C	109.5
C10—C9—C8	119.5 (4)	H17A—C17—H17C	109.5
C10—C9—H9	120.3	H17B—C17—H17C	109.5
C8—C9—H9	120.3	C13—N1—C1	126.4 (3)
C11—C10—C9	120.7 (3)	C13—N1—H18	118 (2)
C11—C10—Cl1	120.1 (3)	C1—N1—H18	116 (2)
C9—C10—Cl1	119.3 (3)	C15—O2—C16	116.6 (3)
C10—C11—C12	119.7 (3)		
			
C6—C1—C2—F1	179.4 (3)	C8—C7—C12—C11	1.9 (5)
N1—C1—C2—F1	−0.8 (5)	C14—C7—C12—C11	−173.6 (3)
C6—C1—C2—C3	0.4 (6)	C10—C11—C12—C7	−0.6 (6)
N1—C1—C2—C3	−179.8 (4)	N1—C13—C14—C15	1.0 (6)
F1—C2—C3—C4	−179.0 (3)	N1—C13—C14—C7	−179.7 (3)
C1—C2—C3—C4	−0.1 (6)	C12—C7—C14—C13	44.5 (5)
C2—C3—C4—C5	0.3 (6)	C8—C7—C14—C13	−130.9 (4)
C2—C3—C4—F2	179.6 (3)	C12—C7—C14—C15	−136.2 (3)
F2—C4—C5—C6	179.8 (4)	C8—C7—C14—C15	48.4 (5)
C3—C4—C5—C6	−0.9 (6)	C13—C14—C15—O1	3.8 (6)
C4—C5—C6—C1	1.2 (6)	C7—C14—C15—O1	−175.5 (4)
C2—C1—C6—C5	−1.0 (5)	C13—C14—C15—O2	−174.1 (3)
N1—C1—C6—C5	179.2 (4)	C7—C14—C15—O2	6.5 (5)
C12—C7—C8—C9	−1.3 (6)	C14—C13—N1—C1	−175.8 (4)
C14—C7—C8—C9	174.2 (3)	C2—C1—N1—C13	−177.2 (3)
C7—C8—C9—C10	−0.5 (6)	C6—C1—N1—C13	2.6 (6)
C8—C9—C10—C11	1.9 (6)	O1—C15—O2—C16	2.9 (5)
C8—C9—C10—Cl1	−177.9 (3)	C14—C15—O2—C16	−179.2 (3)
C9—C10—C11—C12	−1.3 (6)	C17—C16—O2—C15	180.0 (3)
Cl1—C10—C11—C12	178.5 (3)		

### Hydrogen-bond geometry (Å, °)

**Table:**

*D*—H···*A*	*D*—H	H···*A*	*D*···*A*	*D*—H···*A*
N1—H18···F1	0.83 (3)	2.29 (3)	2.674 (3)	108 (3)
N1—H18···O1	0.83 (3)	2.07 (3)	2.675 (4)	129 (3)
C6—H6···O1^i^	0.93	2.51	3.321 (4)	146

Symmetry codes: (i) *x*, −*y*+3/2, *z*−1/2.
